# Stress Monitoring and Recent Advancements in Wearable Biosensors

**DOI:** 10.3389/fbioe.2020.01037

**Published:** 2020-09-02

**Authors:** Cheyenne Samson, Ahyeon Koh

**Affiliations:** Department of Biomedical Engineering, Thomas J. Watson School of Engineering, Binghamton University, Binghamton, NY, United States

**Keywords:** stress biomarkers, stress sensors, wearable cortisol sensors, skin-interfaced cortisol sensors, stress analysis and evaluation

## Abstract

The stress response allows the body to overcome obstacles and prepare for threats, but sustained levels of stress can damage one’s health. Stress has long been measured through physical tests and questionnaires that rely primarily on user-inputted data, which can be subjective and inaccurate. To quantify the amount of stress that the body is experiencing biologically, analytical detection of biomarkers associated with the stress response recently have been developed. Novel stress sensing devices focus on cortisol sweat sensing as a part of wearable, flexible devices. These devices promise a real-time, continuous collection of stress data that can be used in clinical diagnoses or for personal stress monitoring and mediation.

## Introduction

Stress was first defined by in 1936 by Hans Selye, a pioneering endocrinologist from Hungary (Rochette and Vergely, 2017), as: “the non-specific response of the body to any demand.” (Fink, 2009). Selye’s subsequent stress experiments started the conversation about stress and its effect on the body (Rochette and Vergely, 2017). Selye’s definition was quite narrow, placing a clear emphasis on only the biological aspects of stress. This has led to different definitions being used depending on context – behavioral scientists define stress as the perception of threat with resulting anxiety or discomfort (Fink, 2009), while neuroendocrinologists define it as any stimulus that triggers the secretion of the adrenocorticotropic hormone and glucocorticoids (Miller and O’Callaghan, 2002). In the context of this manuscript, stress is defined as any event that disrupts homeostasis, resulting in the release of hormones to return the body to homeostasis.

**FIGURE 1:**
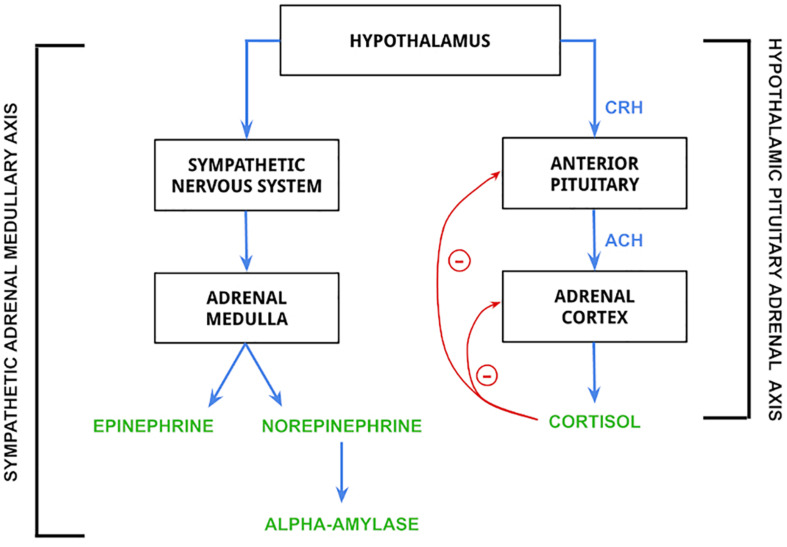
Mechanism of release for epinephrine, norepinephrine, alpha-amylase, and cortisol. Biomarkers in the figure are controlled by the hypothalamus. The hypothalamic pituitary adrenal (HPA) axis is activated by hypothalamic release of corticotropin-releasing hormone (CRH), which stimulates the secretion of adrenocorticotropin hormone (ACH) from the anterior pituitary gland. ACH then acts on the adrenal cortex to stimulate the release of cortisol (Lanoix and Plusquellec, 2013; Antoun et al., 2017), which is regulated by negative feedback loops to both the anterior pituitary and adrenal cortex. The sympathetic adrenal medullary (SAM) axis is activated by the hypothalamus to stimulate a sympathetic nervous system response, which then stimulates the adrenal medulla to produce epinephrine and norepinephrine (Antoun et al., 2017). The release of norepinephrine is then responsible for the synthesis and release of alpha-amylase (Sawami et al., 2017).

Stress is biologically associated with several disorders and related health problems. The industry standard for diagnosis of mental disorders (DSM-V) recognizes two stress-related disorders: Acute Stress Disorder (ASD) and Post Traumatic Stress Disorder (PTSD) (Fink, 2009; Bakhshian et al., 2013). Biologically, ASD and PTSD are associated with increased levels of cortisol and abnormal function of the hypothalamic-pituitary-adrenal (HPA) axis while being very different in their psychological severity (Bakhshian et al., 2013; Marin et al., 2019). The HPA axis works concurrently with the sympathoadrenal-medullary (SAM) axis of the sympathetic nervous system to stimulate the release of several hormones that prepare the body to survive a stressful situation. These axes are also instrumental in the appropriate termination of the release of stress hormones to maintain homeostasis and proper bodily function (Murison, 2016), and their effects can be visualized in Figure 1 below. In addition to PTSD and ASD, increased stress levels have been linked to decreased cardiovascular health (Poirat et al., 2018) and increased risk of anxiety-depressive symptoms (Marcatto et al., 2016).

The objective of this manuscript is to provide a comprehensive review of stress, what causes stress, and what methods and technologies are being used to measure stress.

## What Makes Us Stressed?

Stress is experienced in a wide range of situations, including familial pressures, personal finances, academics, and more (Fairbrother and Warn, 2003; Reddy et al., 2018). Any stimulus that causes a stress response is a stressor (Schneiderman et al., 2005), which is defined as any environmental change that causes a shift toward a state of lower utility (Oken et al., 2015). In other words, a stressor is anything that causes a homeostatic imbalance and results in a biological or behavioral reaction to correct this imbalance (Oken et al., 2015; Murison, 2016). This imbalance, termed the stress response, varies in severity and duration from person to person (Oken et al., 2015). Homeostasis is the body’s innate and dynamic ability to make physiological changes to maintain an adequate environment to perform all necessary physiological functions, and this definition has been the dominant explanation of self-regulation since it was coined by Walter Cannon in 1929 (Ramsay and Woods, 2014). Homeostasis, however, is a broad term, so the term allostasis is used to describe the physiological changes the body makes specifically in response to a stressor to maintain physiological balance (Schulkin and Sterling, 2019). According to allostatic principles, anticipation of a possible stressful event followed by appropriate regulation by the brain is the best way to physiologically regulate one’s stress response (Ramsay and Woods, 2014). Therefore, the allostatic load that an individual experiences is highly variable, as the ability or inability to anticipate stressful events also varies based on the individual (Szalma, 2008; Schulkin and Sterling, 2019).

Evidence exists correlating neonatal experiences to the level of stress hormones released later in life (Champagne et al., 2003; Schneiderman et al., 2005). Champagne et al. (2003) showed that rats raised by nurturing mothers produced higher levels of serotonin, the happy hormone, later in their lives. It has been shown that serotonin is effective in suppressing the hormones related to panic, which is directly related to the stress response (Deakin and Graeff, 1991; Hood et al., 2006). Additionally, those rodent models raised by nurturing mothers, in turn, were great nurturers of their young (Champagne et al., 2003). In humans, childhood neglect has been shown to alter and shape the development of the HPA axis, the key controlling system of the stress reaction (Reilly and Gunnar, 2019).

While how each individual interprets stressors and reacts is highly variable, the biological response to stress is controlled by the HPA axis, which is responsible for the controlled release of hormones such as cortisol, ACTH, adrenaline, and noradrenaline (Murison, 2016). These hormones work together to give the body the best chance of survival against the perceived threat that a stressor poses (Oken et al., 2015). Indeed, stress-related disorders are psychological and caused by traumatic events but can be characterized biologically with this increased expression of cortisol within the body (Marin et al., 2019). Other factors that can influence HPA axis reactivity, and therefore the magnitude of the stress response, are age, sex, genetics, and prescription drugs (Zänkert et al., 2019).

## How to Measure Stress

### Clinical Tests

Several behavioral tests exist to quantify stress levels experienced by an individual (Oken et al., 2015). The Trier Social Stress Test (TSST) is commonly used to quantify acute stress by having the subject first perform a public speaking test followed by an arithmetic calculation. After these tasks are performed, analyses of the subject’s saliva, blood, psychophysiological, and cognitive measures are made to assess stress levels (Allen et al., 2014). However, TSST is not perfect because of the great variability between room set-up, timing of events, etc. from trial to trial. This leads to inconsistencies and the inability to reproduce results using TSST (Labuschagne et al., 2019). Further efforts are being made to standardize TSST and other similar tests to make the results more reliable and reproducible (Dedovic et al., 2005; Labuschagne et al., 2019).

The Perceived Stress Scale (PSS) is a tool used by medical professionals to assess an individual’s overall stress levels before any physical or psychological intervention (Nielsen et al., 2016). PSS is a simple survey that asks an individual several questions regarding the past month of their life on a scale from 0 (never) to 4 (very often). Examples of the questions that are asked are “How often have you been upset because of something that happened unexpectedly?” and “How often have you felt that things were going your way?” (Hewitt et al., 1992). These questions are meant to quantify the extent to which a subject has perceived their life as “unpredictable, uncontrollable, and overloading” in the past month (Nielsen et al., 2016), and therefore give insight into their stress level.

Structured similarly to PSS, the Kessler Psychological Distress Scale (K10) uses a set of 10 questions to quantify the level of mental distress a person is experiencing. These questions are answered based on the past month on a scale from 1 (none of the time) to 5 (all of the time) and include those such as “Did you feel nervous?” and “Did you feel depressed?.” These questions were specifically curated to target depressive, anxious, and other psychologically disturbing feelings to assess the mental health levels of individuals. K10 has proven to be precise and reliable and allows for accurate determination of DSM-V cases and non-cases (Kessler et al., 2002).

### Biomarkers

Stress can be quantified by measuring levels of different stress biomarkers that exist in bodily fluids. A biomarker is a molecule or other indicator that can give insight into the health of an individual via *ex vivo* analysis. Biomarkers provide quantifiable measures of biological processes, which gives medical professionals the ability to investigate issues happening within the body without having to perform a surgical procedure (Strimbu and Tavel, 2011). Non-invasive sampling of biofluids such as saliva and sweat provides the opportunity for continuous and real-time monitoring of analytes (Katchman et al., 2018), as well as providing a convenient and easy collection process for patient and collector. Some stress biomarkers, like cortisol, are present in more than one biofluid and cross-analysis can help validate results. Biomarkers are released and classified by two main systems in the body: hormones released by the endocrine system and neurotransmitters released by the nervous system (Steckl and Ray, 2018). The system that controls the release of the biomarker influences what biofluid it is present in. The main indicators of a stress response include dopamine, epinephrine, norepinephrine, serotonin, alpha-amylase, cortisol, and interleukin-6 (Goldstein, 2010; Ranabir and Reetu, 2011; Steckl and Ray, 2018; Dhama et al., 2019; Gug et al., 2019). Each one of these molecules plays a specific role in the stress response; all working to activate systems to ready the body to overcome the stressor evoking the response.

#### Epinephrine and Norepinephrine

Epinephrine and norepinephrine are hormones secreted by the adrenal gland that are vital in the evocation and regulation of the fight-or-flight response (Steckl and Ray, 2018). They cause increases in heart and respiratory rate, as well as suppress the immune system to shunt energy toward vital physiological systems to ready the body to respond and survive a perceived threat (Sanchez et al., 2003; Breed and Moore, 2012). Epinephrine and norepinephrine can be found in the blood at concentrations of 0–0.028 and 0.06 ng/mL, respectively. They are also present in the urine at concentrations of 0–20 and 15–80 ng/mL, respectively (Steckl and Ray, 2018).

#### Alpha-Amylase

Alpha-amylase is an enzyme that cleaves large alpha-linked polysaccharides into glucose and maltose to be used as immediate energy sources. While alpha-amylase is not technically a biomarker, literature in recent years has shown that high levels of salivary alpha-amylase may indicate chronic stress (Nater and Rohleder, 2009; Vineetha et al., 2014; Steckl and Ray, 2018; Ali and Nater, 2020). The confirmation of salivary alpha-amylase as a reliable biomarker for stress will allow for greater variability in stress quantification approaches through saliva samples. Alpha-amylase is present in the saliva at concentrations of 0.6–2.6 mg/mL (Nater and Rohleder, 2009). As seen in Figure 1, alpha-amylase release is controlled by the SAM axis, and the co-activation patterns between SAM and HPA axes is being studied as indications of a stress response (Wadsworth et al., 2020).

#### Cortisol

Cortisol is a glucocorticoid hormone, whose release is controlled by the central nervous system, more specifically the HPA axis. Cortisol is currently considered the gold standard for evaluating the activity of the HPA axis (Ali and Nater, 2020). Glucocorticoids are responsible for reallocation of energy to overcome real or anticipated stressors perceived during stress response (Herman et al., 2016). Cortisol synthesis and release are controlled by adrenocorticotropic hormone (ACTH), which is regulated by the levels of corticotropin-releasing hormone (CRH) by the hypothalamus (Katsu and Iguchi, 2015). Cortisol fluctuates cyclically with the circadian rhythm, with concentrations peaking in the morning and decreasing throughout the 12-h day (Rao and Androulakis, 2019). Cortisol binds to intracellular receptors to reduce inflammation, maintain blood pressure, suppress the immune system, and manage stress (Katsu and Iguchi, 2015). Cortisol is present in blood, saliva, sweat, urine, and cerebrospinal fluid. Especially, saliva and sweat are currently researched for stress devices because of their reliability and ease-of-collection. The physiological ranges of cortisol in blood, saliva, and sweat are 30–230 ng/mL (Steckl and Ray, 2018), 0.1–10 ng/mL (Dhull et al., 2019), and 8–140 ng/mL (Jia et al., 2016), respectively.

## Recent Developments in Stress Monitoring Systems

Through smartwatches (Hsiao and Chen, 2018), fitness trackers (Lee and Lee, 2018), and the general desire for smart, at-home health services (Wiegard and Breitner, 2019), the general public is arguably more in-tune with their health and history than ever before (Kim and Kim, 2018). Many devices aimed at monitoring real-time stress rely on photoplethysmography data (Park et al., 2018), and some groups have integrated photoplethysmography data with other physiological signals such as heart rate variability (Mohan et al., 2016) or ECG data and respiratory signals (Chen et al., 2015). However, these signals are not the direct cause of a stress response, rather they are the physiological effect that results from the release of stress biomarkers in the body. As such, sensing of biomarkers may provide a more accurate way of stress sensing and will be the main focus of this manuscript. Several stress management applications are available for download on smartphones that provide coping strategies such as breathing, mindfulness, and mediation to help combat a stressful lifestyle. Apps which are evidence based are capable of supplementing medical care, but not at specifically quantifying stress levels (Coulon et al., 2016). To quantify stress more accurately, researchers are developing devices that can give relevant, concrete data through detection of specific stress biomarkers for stress monitoring. The recent developments in novel, non-invasive, wearable (or portable) sweat sensors will be discussed in further detail in next section.

### Salivary Sensors

While salivary detection methods may not be conducive to wearable technologies, that does not mean that they have no place in point-of-care applications. A recent study by Liu et al. (2020) has shown that a portable salivary cortisol detection device can provide information that is just as useful as a wearable one. A portable differential pulse voltammetry (DVP) system was created that can be completely controlled via smartphone to deliver information to the individual about stress status. The working electrode, connected to a printed circuit board, is functionalized via anti-cortisol antibodies being linked to a gold nanoparticle electrode surface. An individual with this system can initiate a cortisol reading through the application, which then communicates to the DVP system to start a scan, which in turn relays the cortisol concentration back to the application via Bluetooth. This system has an acceptable linear range of 0.5–200 nM, or 0.18–72.5 ng/mL (Liu et al., 2020). While the ability to have a wireless connection to a smartphone is extremely advantageous, this type of system does not allow for real-time analysis. Research into salivary alpha-amylase for stress assessment is also being done and a protype for handheld monitoring has been created (Hsiao et al., 2019). Given that salivary alpha-amylase does not fluctuate with the circadian rhythm, as cortisol does, more emphasis on this area may be seen in the future. Nevertheless, the developments discussed further in this manuscript focus on cortisol, as its measurement is the most promising at this time.

### Sweat Cortisol Sensors

Development and investigation of wearable sweat sensors has increased 10-fold in recent years. While the clinical relevance of biomarkers in sweat holds great potential, research is striving to determine the relevance for health monitoring (Chung et al., 2019). Devices being developed for cortisol analysis are validated with enzyme-linked immunosorbent assay (ELISA), an assay that uses antibodies coupled with an enzyme-mediated color change for colorimetric detection of an antigen. ELISA kits are widely available for purchase commercially for various antibodies and the biofluids they exist in (Gan and Patel, 2013).

**FIGURE 2:**
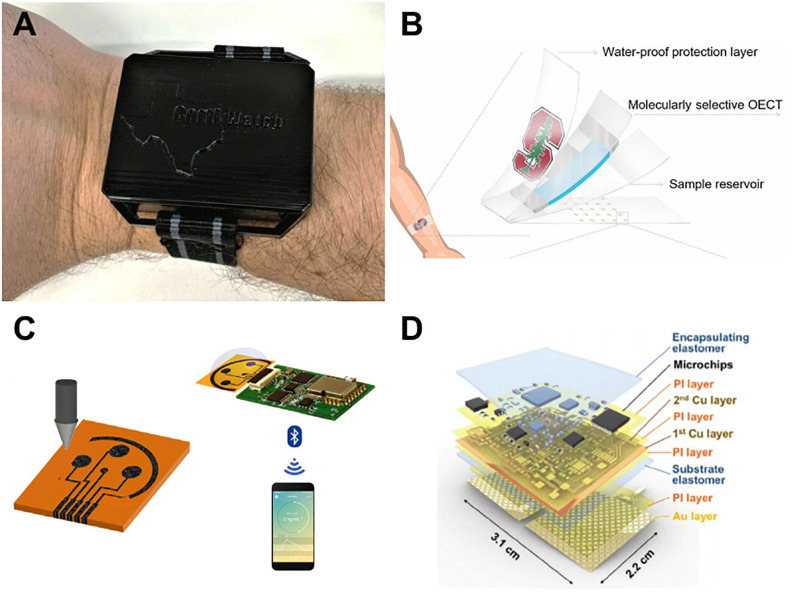
Recent sweat stress sensor designs. **(A)** Wearable CortiWatch (Rice et al., 2019) reprinted via Creative Commons Attribution 4.0, **(B)** MIP sensor on flexible SEBS substrate (Parlak et al., 2018) reprinted via Creative Commons Attribution 4.0. **(C)** printed graphene-based wireless cortisol sensor, reprinted with permission from Torrente-Rodríguez et al., copyright 2020 by Elsevier (Torrente-Rodríguez et al., 2020). **(D)** galvanic skin response sensor SKINTRONICS (Kim et al., 2020) reprinted via Creative Commons Attribution 4.0.

Electrochemical detection methods are commonly used because of their ability to transduce biological signals into electrical ones through functionalized electrodes (Cho et al., 2020). One of the more common ways to functionalize an electrode is by taking advantage of the specificity of an antibody-antigen link, which uses the same theory behind ELISA detection. Figure 2 shows highlighted wearable devices for monitoring stress. A wearable, watch-like device “CortiWatch” has developed by Rice et al. (2019) that uses alpha-cortisol antibody-antigen detection as the functional sensor of chronoamperometric cortisol sensing unit. The working electrode was fabricated by immobilizing alpha-cortisol antibodies onto a gold electrode surface, which was circuited to a potentiostatic robust circuit board to collect data (Rice et al., 2019). While this device is wearable with all electronics housed in a 3D printed box, as shown in Figure 2A, it does not conform perfectly to the skin surface, which can leave gaps in sample collection and analysis. Another flexible wrist-worn device has been developed by Kinnamon et al. (2017) that overcomes this dilemma by integrating a flexible electrode system into their device. The electrode system contains molybdenum disulfide nanosheets functionalized with alpha-cortisol antibodies that allow for a dynamic sensing range of 1–500 ng/mL. This system also shows the ability to perform real-time analysis of cortisol, given that it takes only 3 min to perform a full frequency sweep for analysis (Kinnamon et al., 2017).

### Wearable Sensor Technologies for Stress Monitoring

To move past rigid circuitry and electronics for wearable devices, many groups are opting to create soft, flexible sensors that can conform to the skin surface. A device developed by Lee et al. (2020) used cortisol-specific MX210 antibodies immobilized on a gold nanostructured surface to fabricate the working electrode in their design. The high density of antibodies that they were able to achieve optimized the sensitivity of their design and created a lower limit of detection of 1 pg/mL and dynamic range of 1 pg/mL–1 μg/mL. This working electrode was a part of a flexible, wearable lab-on-a-patch platform developed with polydimethylsiloxane that used microfluidic sweat collection with detection via redox mediator reagent reaction and faradaic electrochemical impedimetric spectroscopy, as shown in Figure 2B; Lee et al. (2020). While using an antibody-antigen detection system is adequate, the sensor has the limitation of antibody-antigen instability and irreproducibility (Schonbrunn, 2014). To overcome this instability, an artificial molecularly imprinted polymer (MIP) has been developed by Parlak et al. (2018) The MIP was synthesized via copolymerization of a functional monomer with a cross-linker in the presence of cortisol – making a template for the cortisol binding sites. The cortisol was then eluted from the substrate, resulting in cortisol-specific binding sites across the MIP surface. Selectivity testing was performed in the presence of cortisol analogs including progesterone, cortisone, and testosterone to find that this novel MIP was adequately selective to measure cortisol concentration without significant interference from unwanted binding. The MIP was found to be reversible, reproducible, and reusable, effectively overcoming the shortcomings that antibody-antigen devices possess, while also being flexible and wearable due to development on a soft styrene-ethylene-butylene-styrene elastomer, as shown in Figure 2C; Parlak et al. (2018). Apart from cortisol sensing, Kim et al. (2020) reported a device they term “SKINTRONICS” which utilizes electrodermal sensing of galvanic skin response paired with temperature recording to determine stress levels. This flexible hybrid electronic device is skin-conformant, accurate, sensitive, and allows for the real-time capture of stress related data. As seen in Figure 2D, SKINTRONICS is a multilayered device with a wear time of up to 7 h. The group was able to achieve galvanic skin response results comparable to those of commercially available devices with the same purpose, but with the added advantage of offering long-term, hands-free use (Kim et al., 2020). Other skin-interfaced sensing platforms are in development, indicating a trend toward flexible sensing (Sekar et al., 2019; Kim et al., 2020).

Bluetooth and other wireless data transfers are highly preferable in sensors that may eventually be used for personal use, as the typical person does not have access to complex computer systems that may be needed to visualize and interpret data. Torrente-Rodríguez et al. (2020) have proposed a device that seemingly contains all key features of a significant sweat-sensing device that is flexible, wearable, accurate, and provides wireless data transmission to a smartphone via Bluetooth. The group has made use of a printed 3 graphene electrode system with an integrated Bluetooth module and microfluidic sample collection. The user is to initiate measurement via Bluetooth, which then triggers the electrode system through a potentiostat interface circuit and provides cortisol measurement in minutes, proving that this design may be applicable for real-time analysis (Torrente-Rodríguez et al., 2020). This design touts the ultimate all-in-one sensing platform which is totally wireless and integrated into a system that allows for an extremely user-friendly interface.

## Conclusion and Future Directions

Sweat sensing is at the forefront of wearable stress detection devices currently in development. These devices show great promise to quantify several sweat biomarkers, namely cortisol, to monitor the levels of stress that an individual is experiencing, as opposed to current stress mediating devices that rely on user input of data (Coulon et al., 2016). This biological information may be useful to supplement medical professional diagnoses of psychological disorders, as well as conditions such as Addison’s and Cushing’s diseases, which are characterized by low and high levels of basal cortisol, respectively (Ragnarsson, 2020; Saverino and Falorni, 2020). Future development of these devices may provide a direct user output instead of relying on additional components to visualize and analyze the data, similar to the attempts made by Liu et al. and Rodríguez et al. This may be achieved via the integration of near-field communication chips for remote data visualization via Bluetooth, which have been tested for flexibility (Jeong et al., 2017). Additionally, further research into other indicators of stress, such as alpha-amylase, may see more attention as a measure of SAM axis activity (Hsiao et al., 2019). The multimodal wearable stress sensors will advance quality-of-life sensing systems that provide accurate, reliable, and viable physiological status enabling to apply artificial intelligences.

## Author Contributions

CS and AK conducted a literature review and wrote the manuscript. Both authors contributed to the article and approved the submitted version.

## Disclaimer

The views and conclusions contained herein are those of the authors and should not be interpreted as necessarily representing the official policies or endorsements, either expressed or implied, of Air Force Research Laboratory, the U.S. Government, or SEMI-FlexTech.

## Conflict of Interest

The authors declare that the research was conducted in the absence of any commercial or financial relationships that could be construed as a potential conflict of interest.
